# Trends in overweight and obesity over 22 years in a large adult population: the HUNT Study, Norway

**DOI:** 10.1111/cob.12009

**Published:** 2013-03-19

**Authors:** K Midthjell, C M Y Lee, A Langhammer, S Krokstad, T L Holmen, K Hveem, S Colagiuri, J Holmen

**Affiliations:** 1 Department of Community Medicine and General Practice, HUNT Research Centre, Norwegian University of Science and TechnologyLevanger, Norway; 2 The Boden Institute of Obesity, Nutrition, Exercise & Eating Disorders, University of SydneySydney, Australia

**Keywords:** Gender differences, Norway, obesity, overweight

## Abstract

Some reports indicate that the obesity epidemic may be slowing down or halting. We followed body mass index (BMI) and waist circumference (WC) in a large adult population in Norway (*n* = 90 000) from 1984–1986 (HUNT1) through 1995–1997 (HUNT2) to 2006–2008 (HUNT3) to study whether this is occurring in Norway. Height and weight were measured with standardized and identical methods in all three surveys; WC was also measured in HUNT2 and HUNT3. In the three surveys, mean BMI increased from 25.3 to 26.5 and 27.5 kg m^−2^ in men and from 25.1 to 26.2 and 26.9 kg m^−2^ in women. Increase in prevalence of obesity (BMI ≥ 30 kg m^−2^) was greater in men (from 7.7 to 14.4 and 22.1%) compared with women (from 13.3 to 18.3 and 23.1%). In contrast, women had a greater increase in abdominal obesity (WC ≥ 102 cm for men and WC ≥ 88 cm for women). There was a continuous shift in the distribution curve of BMI and WC to the right, demonstrating that the increase in body weight was occurring in all weight groups, but the increase of obesity was greatest in the youngest age groups. Our data showed no signs of a halt in the increase of obesity in this representative Norwegian population.

## What is already known about this subject

- Prevalence of overweight and obesity has been increasing worldwide, but recent reports suggest the trend in some countries may be plateauing
- Prevalence of overweight and obesity derived from body mass index can be different to that derived from waist circumference.

## What this study adds

- Obesity was more prevalent and increased more when defined by waist circumference than by body mass index.
- While the increase in obesity prevalence as defined by body mass index was greater in men, the increase in obesity prevalence as defined by waist circumference was greater in women.
- The increase in prevalence for body mass index-defined overweight was significant in both sexes over the first half of the period studied, but was only significant in men over the second half.

## Introduction

The prevalence of overweight and obesity is increasing worldwide 1,2; although some recent reports indicate that the obesity epidemic may be slowing down, especially in children and adolescents 3–10. We have previously reported a considerable increase in obesity in Norway between 1984–1986 and 1995–1997, coinciding with an increase in the prevalence of diabetes 11. The health consequences of overweight and obesity are well documented, irrespective of whether it is expressed as general obesity by body mass index (BMI) 12–14 or as abdominal obesity by waist circumference (WC) 15–19. Both contribute separately to increased morbidity 20 and mortality 16.

The Nord-Trøndelag Health Study (The HUNT Study) has followed a large, non-selected adult population in central Norway through three cross-sectional surveys during a 22-year period, enabling the study of longitudinal changes in weight measures in adults aged 20–99 years.

## Subjects and methods

### Population

Nord-Trøndelag County is located in the middle part of Norway at 64 degrees North, with a population of 127 000 in 1984, increasing to 130 000 in 2008. The population structure of Nord-Trøndelag is stable and fairly representative of Norway, except that it has no big city (the largest town having 20 500 inhabitants), mean income and education level is slightly less than the national average, and foreign immigration is lower than in the more densely populated areas of Norway. Although immigration from other cultures increased somewhat over the years and there is a small Lappish (Sami) population, the population studied is almost exclusively Caucasian (around 97% in 2000 21,22).

### Data collection

#### HUNT1

In the first HUNT survey (HUNT1, 1984–1986), all inhabitants in the county aged 20 years and over were invited, and 74 436 individuals (87%) participated in a clinical examination and had their height and weight measured 11,23. Height was measured without shoes to the nearest centimetre. Weight was measured with light clothes, without shoes, jacket or outdoor garments to the nearest half kilogram. We excluded 2354 participants who either were not able or willing to have their height or weight measured, or reported to be pregnant. Participants answered extensive health-related questionnaires.

#### HUNT2

The second survey (HUNT2) was conducted in 1995–1997. All inhabitants aged 13 years or more were invited 21. Of those aged 20 years and over, 64 804 individuals (70%) attended the clinical examination and had their height and weight measured by the same method as in HUNT1. After excluding 2162 participants who could not be measured or were pregnant, a total of 62 642 were included in the analysis. Nearly all participants (*n* = 62 513) also had their WC and hip circumference measured to the nearest centimetre by applying a non-stretchable band with the participant standing relaxed with arms on the side. WC was measured horizontally at the umbilical level, and hip circumference at the thickest part of the hips.

#### HUNT3

The third survey (HUNT3) was conducted from 2006 to 2008, again addressing all inhabitants aged 13 years or more 24. In all, 50 386 participants (54%) aged 20 years and over participated in the clinical examination and had their height, weight, WC and hip circumference measured using the same protocol as in HUNT2. Five hundred fifty-seven participants were excluded from the analysis because of pregnancy or because height and/or weight could not be measured. Of the 49 829 participants with valid height and weight data, 49 739 also had their WC measured.

### Ethics

All participants in the three HUNT surveys consented according to Norwegian law and recommendations; in HUNT1 by informed and voluntary participation, in HUNT2 and HUNT3 by signed informed consent. Approvals were obtained from the Regional Committee for Ethics in Medical Research and the Norwegian Data Inspectorate.

### Statistics

Included in the analyses were BMI measured in all three surveys and WC in HUNT2 and HUNT3. BMI was calculated as weight in kg/(height in m)^2^. According to the World Health Organization, overweight and obesity were defined as BMI 25–29.9 kg m^−2^ and BMI ≥ 30 kg m^−2^, respectively 1,25. Obesity was further classified into classes I, II and III for those with BMI 30–34.9 kg m^−2^, BMI 35–39.9 kg m^−2^ and BMI ≥ 40 kg m^−2^, respectively 1. Abdominal obesity was defined as WC ≥ 102 cm for men and WC ≥ 88 cm for women, equivalent to ‘significantly increased risk’ in a Caucasian population. Correspondingly, abdominal overweight was defined as WC 94–101.9 cm for men and WC 80–87.9 cm for women, equivalent to ‘increased risk’ in this type of population 1,25–29. The central obesity criterion for Europids used in the International Diabetes Federation's definition for the metabolic syndrome 30,31 is equivalent to the sum of abdominal overweight and obesity (WC ≥ 94 cm for men and ≥ 80 cm for women) by the World Health Organization definition used here.

Prevalence of BMI-based overweight and obesity and abdominal overweight and obesity are reported by sex and 10-year age groups. Two-sample *z*-test was used to compare mean values and two-proportion *z*-test was used to compare prevalence between HUNT1 and HUNT2 and between HUNT2 and HUNT3. Statistical significance was set at *P* < 0.05. SAS 9.1 for Windows (SAS Institute, Inc., Cary, NC, USA) was used for the analysis.

## Results

Table 1 shows the characteristics of participants in the three surveys. More women than men participated in HUNT2 and HUNT3. HUNT3 participants were generally older and taller than HUNT1 and HUNT2 participants. In men, mean BMI increased from 25.3 kg m^−2^ in HUNT1 to 27.5 kg m^−2^ in HUNT3. The corresponding increase in women was from 25.1 to 26.9 kg m^−2^. Mean WC increased from 91.9 to 97.4 cm in men and from 81.4 to 90.3 cm in women. Hip circumference also increased significantly from HUNT2 to HUNT3.

**Table 1:** Characteristics of participants in HUNT1, HUNT2 and HUNT3

	HUNT1 (1984–86)		HUNT2 (1995–97)		HUNT3 (2006–08)	
			
	Men	Women	Men	Women	Men	Women
N	35440	36642	29646	32996	22658	27171
Mean age (years)	48.1 (16.9)	49.1 (17.3)	49.3 (16.5)*	49.8 (17.0)*	53.7 (15.4)∧	52.9 (16.1)∧
Mean (SD) height (cm)	176.3 (6.7)	163.0 (6.3)	177.3 (6.8)*	163.9 (6.4)*	177.8 (6.7)∧	164.6 (6.3)∧
Mean (SD) BMI (kg m^−2^)	25.3 (3.2)	25.1 (4.5)	26.5 (3.5)*	26.2 (4.6)*	27.5 (3.8)∧	26.9 (4.9)∧
Mean (SD) waist circumference (cm)	–	–	91.9 (9.3)	81.4 (11.4)	97.4 (10.5)∧	90.3 (12.7)∧
Mean (SD) hip circumference (cm)	–	–	102.3 (6.2)	101.9 (9.5)	103.6 (6.5)∧	103.8 (9.2)∧
Mean (SD) waist-to-hip ratio	–	–	0.9 (0.05)	0.8 (0.06)	0.9 (0.07)	0.9 (0.07)∧
Overweight (BMI 25–29.9 kg m^−2^) (%)	42.1	29.9	50.5*	37.1*	52.4∧	37.7
Obese class I (BMI 30–34.9 kg m^−2^) (%)	6.8	10.1	12.5*	13.7*	18.5∧	16.6∧
Obese class II (BMI 35–39.9 kg m^−2^) (%)	0.8	2.6	1.7*	3.6*	3.2∧	5.0∧
Obese class III (BMI ≥ 40 kg m^−2^) (%)	0.1	0.7	0.2*	1.0*	0.5∧	1.5∧
Total overweight (BMI ≥ 25 kg m^−2^)	49.8	43.2	64.9*	55.4*	74.5∧	60.8∧
Total obesity (BMI ≥ 30 kg m^−2^)	7.7	13.3	14.4*	18.3*	22.1∧	23.1∧
Abdominal overweight (WC M: 94–101.9 cm, F: 80–87.9 cm) (%)	–	–	25.9	24.5	31.9∧	23.7∧
Abdominal obesity (WC M: ≥ 102 cm, F: ≥ 88 cm) (%)	–	–	13.7	26.9	31.9∧	55.9∧

* *P* < 0.05 between HUNT1 and HUNT2.

∧ *P* < 0.05 between HUNT2 and HUNT3.

BMI, body mass index; SD, standard deviation; WC, waist circumference.

**Figure 1:**
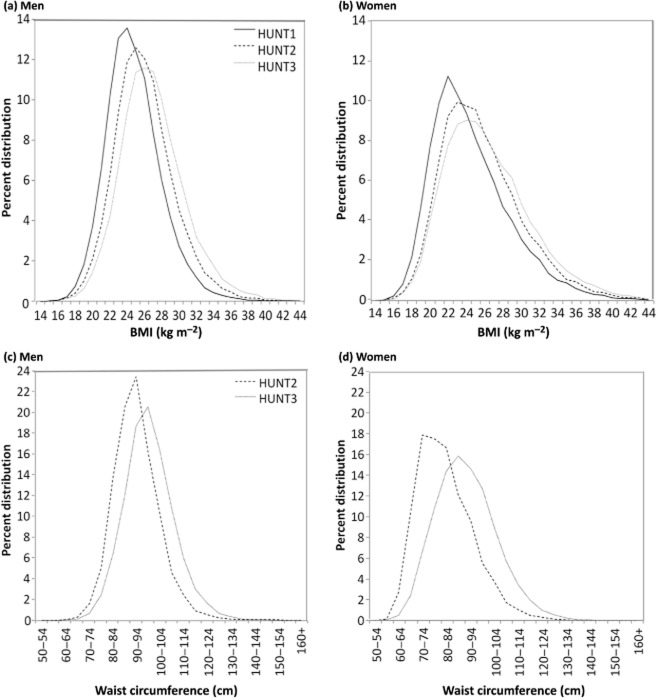
Distribution of body mass index (BMI) in (a) men and (b) women in HUNT1, HUNT2 and HUNT3 and of waist circumference in (c) men and (d) women in HUNT2 and HUNT3 expressed as percentage distribution.

The distribution curve for BMI in HUNT2 was broader, with a lower peak, and moved to the right of that in HUNT1 (Fig. 1). This rightward shift continued in HUNT3. The distribution curve for WC in HUNT3 was lower and shifted rightward compared with the HUNT2 curve in both sexes, but more in women than men (Fig. 1). The rightward shift in distribution was greater for WC than for BMI. The rightward shift in BMI from HUNT2 to HUNT3 was greater in men, while the rightward shift for WC was greater in women in accordance with the mean values in Table 1.

### Change in overweight

The prevalence of BMI-defined overweight increased from 42.1% in HUNT1 to 52.4% in HUNT3 in men, and from 29.9 to 37.7% in women (Table 2). The increase was largest from HUNT1 to HUNT2 in both sexes. No significant increase from HUNT2 to HUNT3 was observed in women.

**Table 2:** Prevalence (%) of overweight (BMI 25–29.9 kg m^−2^) and obesity (BMI ≥ 30 kg m^−2^) by sex and age group

Age group	HUNT1			HUNT2			HUNT3		
			
	*n*	Overweight	Obese	*n*	Overweight	Obese	*n*	Overweight	Obese
Men									
20–29	5853	25.0	3.7	3905	37.0*	8.4*	1739	35.4	13.3∧
30–39	7941	38.5	5.5	5360	49.1*	12.7*	2837	51.5∧	21.3∧
40–49	5917	46.0	8.0	6461	53.1*	14.4*	4534	54.2	23.5∧
50–59	5517	49.7	9.7	5332	54.6*	17.8*	5384	55.2	23.5∧
60–69	5862	49.2	10.7	4241	54.0*	17.4*	4625	54.7	24.6∧
70–79	3469	48.4	10.6	3436	52.2*	15.9*	2604	53.3	21.3∧
80+	881	42.1	8.9	911	51.6*	10.7	935	48.5	15.2∧
20+	35440	42.1	7.7	29646	50.5*	14.4*	22658	52.4∧	22.1∧
Women									
20–29	5767	14.6	3.7	4433	26.7*	10.1*	2414	25.0	13.6∧
30–39	7957	19.0	6.0	5881	30.5*	11.7*	3917	31.0	20.3∧
40–49	5947	28.8	9.7	6990	36.1*	14.5*	5425	37.1	20.5∧
50–59	5552	38.5	16.7	5709	41.9*	20.4*	5966	40.8	23.1∧
60–69	6069	42.1	23.5	4582	44.4*	26.6*	5085	41.7∧	28.2
70–79	4112	41.9	25.4	4074	42.3	29.2*	3027	42.4	30.0
80+	1238	39.1	17.9	1327	43.2*	24.9*	1337	43.8	23.8
20+	36642	29.9	13.3	32996	37.1*	18.3*	27171	37.7	23.1∧

* *P* < 0.05 between HUNT1 and HUNT2.

∧ *P* < 0.05 between HUNT2 and HUNT3.

In men, the increase in overweight from HUNT1 to HUNT2 was significant in all age groups, but was largest among the youngest (Table 2). From HUNT2 to HUNT3, only age group 30–39 years showed a significant increase.

In women, the increase from HUNT1 to HUNT2 was significant in all age groups except age group 70–79 years. Even among women, the change was largest in the youngest age groups (Table 2). From HUNT2 to HUNT3 there was no significant increase of overweight in any age group (Table 2).

### Change in obesity

In men, BMI-based obesity increased from 7.7% in HUNT1 to 22.1% in HUNT3 and in women from 13.3 to 23.1% (Table 2). From HUNT1 to HUNT2 the increase was significant in all age groups for both sexes, except age group 80+ years in men.

In men, the increase from HUNT2 to HUNT3 was of equal size as from HUNT1 to HUNT2 and was equally distributed amongst age groups. The increase was significant in all age groups (Table 2). In women, the increase of obesity from HUNT2 to HUNT3 was largest in the youngest age groups, while the increase was not significant in those aged 60 years and over (Table 2).

### Obesity class I-III

In men, the prevalence of obesity class I increased nearly threefold from 6.8% in HUNT1 to 18.5% in HUNT3 (Table 1), fairly equally distributed among age groups. The same pattern was seen in obesity class II and class III, though only 107 men were in class III.

In women, obesity class I increased from 10.1% in HUNT1 to 16.6% in HUNT3 (Table 1). The increase was largest among younger age groups, while there was no increase from HUNT2 to HUNT3 in the oldest age groups. More women than men were classified as having obesity class II and III, and in women the increase was especially large from HUNT2 to HUNT3 in the youngest age groups.

### Abdominal overweight and obesity

The prevalence of abdominal overweight was higher in men than in women in all age groups except age group 20–29 years, but the prevalence did not change significantly from HUNT2 to HUNT3 (Table 3). In contrast, the prevalence of abdominal obesity increased by 117% over the 11-year period, and the prevalence was substantially higher in women than in men in all age groups. The prevalence of abdominal obesity nearly tripled in women below age 50 years (Table 3).

**Table 3:** Prevalence (%) of abdominal overweight (WC 94.0–101.9 cm for men and 80.0–87.9 cm for women) and obesity (WC ≥102.0 cm for men and ≥88 cm for women) by sex and age group

Age group	HUNT2			HUNT3		
		
	*n*	Overweight	Obese	*n*	Overweight	Obese
Men						
20–29	3902	12.5	5.5	1738	18.2∧	13.9∧
30–39	5352	22.2	8.4	2834	29.6∧	25.1∧
40–49	6453	25.5	11.0	4532	32.6∧	29.6∧
50–59	5325	30.5	15.3	5378	34.8∧	33.4∧
60–69	4234	30.7	20.2	4623	33.0∧	38.5∧
70–79	3428	32.4	23.0	2601	33.8	38.2∧
80+	904	32.9	24.8	932	32.5	38.5∧
20+	29598	25.8	13.7	22638	31.9∧	31.9∧
Women						
20–29	4418	16.6	12.1	2388	25.0∧	32.6∧
30–39	5864	20.7	16.1	3903	26.1∧	46.3∧
40–49	6985	24.0	21.2	5422	26.4∧	51.5∧
50–59	5702	26.5	30.4	5960	24.9	58.7∧
60–69	4576	29.4	38.5	5079	21.1∧	64.6∧
70–79	4058	29.2	45.1	3020	18.4∧	69.1∧
80+	1312	30.6	44.3	1329	20.1∧	68.0∧
20+	32915	24.5	26.9	27101	23.7∧	55.9∧

∧ *P* < 0.05 between HUNT2 and HUNT3.

The greatest increase in nearly all grades of obesity was observed in the 30–39 years age group in both sexes for BMI- and WC-defined obesity.

Additional tables and figures are available at the publisher's web-site (see Supporting Information).

## Discussion

### Main results

Our data demonstrate that the obesity epidemic continued in this fairly representative, almost exclusively Caucasian population in Norway. It is worrying that the greatest increase was seen in the younger adult groups, as the risk of obesity complications increases with duration of obesity 32. An important finding is, however, that the increase in overweight was significantly smaller in the second period (between HUNT2 and HUNT3); 1.9% (from 50.5 to 52.4% in men) and 0.6% correspondingly in women compared with 8.4% in men and 7.2% in women, respectively, in the first period (between HUNT1 and HUNT2). This considerably smaller increase in overweight may give hope of a future reduction in the increase of obesity in Norway in line with studies indicating that the obesity epidemic is already slowing down 3–5, in the United States most pronounced in women 6, but recently also in men 9.

Nevertheless, we observed a shift in the distribution curves of BMI and WC to the right, indicating that the change was not only due to fat people getting fatter. The shift in BMI was considerably greater than that observed for American adults between 1999/2000 and 2007/2008 6. Similar patterns were observed in 40–42-year-old Norwegians from three other counties 33. Additionally, both self-reported 34 and measured 35 data demonstrated that overweight and obesity also increased in children in Norway. In addition to this general shift towards higher body weight in all weight categories, there was an especially large increase in obesity classes II and III even between HUNT2 and HUNT3 in most age groups of men and younger age groups of women.

In the US National Health and Nutrition Examination Surveys 6, which also used measured data, the prevalence of obesity in non-Hispanic white men aged ≥20 years increased from 27.3% in 1999–2000 to 33.1% in 2005–2006 and 31.9% in 2007–2008. In our study, the obesity prevalence in the same age and sex group increased from 14% in 1995–1997 to 22.1% in 2006–2008. Although our study showed lower obesity prevalence than the US comparable group, the sum of overweight and obesity groups in the latest surveys (BMI ≥ 25 kg m^−2^) was similar (HUNT 67.0% vs. the United States 67.5%). In a recent worldwide review, Finucane *et al*. 2 estimated a global increase in mean BMI of 0.4 kg m^−2^ per decade. The corresponding HUNT figures for the 22-year period covered was 1.0 kg m^−2^ per decade compared with 1.1 kg m^−2^ per decade for the United States.

#### Different measures

BMI is the most widely applied measure in epidemiological studies, but it has well-known limitations as a surrogate measure for body fat, especially in those with high muscle mass. Furthermore, it has recently been shown that many people identified with obesity based on body fat measurements had BMI in the normal range 36. WC, reflecting central or visceral obesity, has gained increasing attention. Both BMI and WC are independently associated with cardiovascular risk and risk of death 16, but their relative importance may differ depending on the end point 37 or sex 38. Future health costs may be predicted better by WC than by BMI 39, and WC may precede other cardiovascular risk factors 40. The distribution curves of BMI and WC were comparable in our study. Obesity was more prevalent and increased to a greater extent when defined by WC than by BMI, indicating a considerable increase in abdominal fat. WC is a strong and additional risk factor for type 2 diabetes and all-cause mortality 41, and is more strongly related to mortality than BMI in persons with diabetes 42, raising health-related concerns in our population. The reason for this distinct increase in WC is difficult to establish. Compared with HUNT2, the proportion of HUNT participants reporting at least 30 min of daily physical activity was greater for HUNT3 in all age groups (data not shown). The amount of sitting time has, however, increased between HUNT2 and HUNT3. Sitting time is positively associated with cardiovascular risk factors (Chau J *et al*. personal communication), and one could speculate that sitting time might affect WC more than BMI. Further research is needed to examine this against more precise measures of body and abdominal fat.

#### Gender differences

Although BMI-defined obesity prevalence increased more in men than in women between HUNT2 and HUNT3, the reverse was observed for WC-defined obesity prevalence. Mean WC increased by 8.9 cm in women and 6.5 cm in men over the same period. A similar increase was observed in both sexes in Canada (10.6 and 6.5 cm, respectively) 43, but over a longer period (1981–2007/2009). A greater increase in women is also reported in Finland 44. In the United States, a much smaller increase, with similar increases between the sexes (2.9 cm in men and 3.2 cm in women), was observed between 1988/1994 and 1999/2000 45.

The same gender differences were found in a recent study from Young-HUNT (the adolescent part of the HUNT Study), following changes in BMI and WC in 13–19-year-old participants with normal weight prospectively for 11 years 46. While 8% of girls and 9% of boys developed BMI-defined obesity, 34% of girls and 9% of boys developed WC-defined obesity.

WC is reported to predict all-cause mortality better in women than in men 37, which make the observed change in WC in women a worry. The substantial increase in WC in women could, at least partly, be related to changes in androgen levels in women 47,48 because total fat mass in women correlates with circulating testosterone levels 49. It is well known that abdominal obesity increases among women after menopause, associated with an increased amount of bioavailable testosterone 50, although this would not explain the increase in pre-menopausal women. Testosterone levels were not measured in our study.

#### Strengths and limitations

In contrast to most other studies, the present study includes a large population with wide age span and long follow-up period. Objective standardised measurements of height and weight were performed by trained personnel, and are more reliable than self-reported data used in many studies. Self-report has been shown to overestimate height and underestimate weight (especially in women), both resulting in lower than actual BMI 51,52, although the degree of underestimation varies 53.

A limitation of the study might be the declining participation rate from HUNT1 to HUNT3, introducing a potential selection bias, especially in the younger age groups. Overweight people might be more inclined to participate in the later surveys, but the opposite could also occur because of fear of unwanted comments on their body weight. Non-participation studies in HUNT1 and HUNT2 demonstrated that young people mostly attributed non-attendance to forgetfulness or practical difficulties like being away for school or work. After HUNT3, a two-page questionnaire was sent to all non-attendees and 6923 persons (16%) answered. BMI based on self-reported height and weight in this group was, in all age and sex groups, slightly lower than the measured values in those who attended 54. The mean difference in BMI (0.6 kg m^−2^ in men and 1.1 kg m^−2^ in women) was similar in men, but higher in women to that reported in an Australian study, also performed in 2007–2008 (0.6 vs. 0.7 kg m^−2^ in men and women, respectively) 53. This might imply that the high prevalence of obesity in men in HUNT3 is not due to the lower attendance, particularly in men, observed in HUNT3.

As the Nord-Trøndelag County is mainly rural with mean income and education levels slightly lower than the national average, the comparability to the Norwegian population might be attenuated. The obesity prevalence in the city of Tromsø in 1994–1995 was 9.5% in men and 11.5% in women compared with 14.4 and 18.3%, respectively, in those of similar age group in HUNT2 (1995–1997) 55. Accordingly, less densely populated areas in Nord-Trøndelag County had more obesity than the county's towns (data not shown). Because overweight is inversely related to socioeconomic status 56, this might also have contributed to some overestimation of obesity prevalence in the present study. However, the increasing non-western immigrant population in the major cities, acknowledged to be more prone to obesity, might have led to an underestimation. Obesity in certain age groups in Tromsø nearly doubled, especially in men, from 1994–1995 to 2001 33. Altogether, we have no evidence that these factors in Nord-Trøndelag have changed significantly over the period of time studied. We, therefore, consider that the observed changes in this study are generally relevant to the Norwegian population.

A third potential limitation might be the 11-year span between the surveys; therefore, we cannot be certain about what has happened in-between. Other Norwegian studies indicate that there has been a continuous increase in body weight in both 40–42-year-olds 11,33 and children 35. However, we cannot completely rule out an initial increase between HUNT2 and HUNT3 followed by a plateau in weight increase.

Our study showed that the increase in obesity in this relatively representative Norwegian adult population continued to increase up to 2008. Of particular concern is the considerable increase in young adults. The increase in BMI-defined obesity was greater in men than in women. The increase in abdominal obesity was even greater and present in all age and sex groups, but the increase was greater in women than in men for almost all age groups. A slower increase in the overweight group may indicate a future plateuing of the increase in obesity. Because of the increase in obesity in this Caucasian population, there is concern that the decreasing cardiovascular morbidity and mortality observed in Norway may reverse when the full effect of smoking cessation is achieved, unmasking consequences of the increasing obesity. More extensive and effective prevention strategies are recommended to avoid a future reduction in the public's health.

## Abbreviations

BMI,: body mass index
HUNT: The Nord-Trøndelag Health Study (HelseUndersøkelsen i Nord-Trøndelag)
WC: waist circumference.

## Supporting Information

**Figure S1.** Prevalence of BMI defined (A) overweight (BMI 25–29.9 kg m^−2^) and (B) obesity (BMI ≥ 30 kg m^−2^) in men

**Figure S2.** Prevalence of BMI defined (A) overweight (BMI 25–29.9 kg m^−2^) and (B) obesity (BMI ≥ 30 kg m^−2^) in women

**Figure S3.** Prevalence of obesity (A) class I (BMI 30.0–34.9 kg m^−2^), (B) class II (BMI 35.0–39.9 kg m^−2^) and (C) class III (BMI ≥ 40.0 kg m^−2^) in men

**Figure S4.** Prevalence of obesity (A) class I (BMI 30.0–34.9 kg m^−2^), (B) class II (BMI 35.0–39.9 kg m^−2^) and (C) class III (BMI ≥ 40.0 kg m^−2^) in women

**Figure S5.** Prevalence of (A) abdominal overweight (WC 94.0–101.9 cm) and (B) abdominal obesity (WC ≥ 102.0 cm) in men

**Figure S6.** Prevalence of (A) abdominal overweight (WC 80.0–82.9 cm) and (B) abdominal obesity (WC ≥ 88 cm) in women.

**Table S1.** Number of participants with obese class II (BMI 35–39.9 kg m^−2^) and obese class III (BMI ≥ 40 kg m^−2^) by sex and age group.
