# Supplementary material for: Trends in overweight and obesity over 22 years in a large adult population: the HUNT Study, Norway
Source: Clin Obes. 2013 Mar 19;3(1-2):12–20. doi: 10.1111/cob.12009 (PMC3734732; doi:10.1111/cob.12009)

**Trends in overweight and obesity over 22 years in a large adult population. The HUNT Study, Norway**

Kristian Midthjell^1^, Crystal Man Ying Lee^2^, Arnulf Langhammer^1^, Steinar Krokstad^1^, Turid Lingaas Holmen^1^, Kristian Hveem^1^, Stephen Colagiuri^2^ and Jostein Holmen^1^

^1^ HUNT Research Centre, Department of Community Medicine and General Practice, Norwegian University of Science and Technology, Levanger, Norway. ^2^The Boden Institute of Obesity, Nutrition, Exercise & Eating Disorders, University of Sydney, Sydney, Australia

**Corresponding author**:

Kristian Midthjell

HUNT Research Centre, Department of Community Medicine and General Practice, Norwegian University of Science and Technology, Forskningsvegen 2, N-7600 Levanger, Norway.

kristian.midthjell@ntnu.no.

**Online Appendix**

Table S1: Number of participants with obese class II (BMI 35-39.9 kg/m^2^) and obese class III (BMI ≥40 kg/m^2^) by sex and age group.

| **Age group** | **HUNT1** | | | **HUNT2** | | | **HUNT3** | | |
| --- | --- | --- | --- | --- | --- | --- | --- | --- | --- |
|  | **Total** | **Obese class II** | **Obese class III** | **Total** | **Obese class II** | **Obese class III** | **Total** | **Obese class II** | **Obese class III** |
| **Men** |  |  |  |  |  |  |  |  |  |
| 20-29 | 5853 | 23 | 4 | 3905 | 49 | 9 | 1739 | 38 | 9 |
| 30-39 | 7941 | 42 | 14 | 5360 | 74 | 16 | 2837 | 103 | 23 |
| 40-49 | 5917 | 39 | 6 | 6461 | 107 | 18 | 4534 | 152 | 21 |
| 50-59 | 5517 | 67 | 6 | 5332 | 116 | 16 | 5384 | 160 | 27 |
| 60-69 | 5862 | 57 | 3 | 4241 | 96 | 7 | 4625 | 177 | 22 |
| 70-79 | 3469 | 33 | 2 | 3436 | 51 | 5 | 2604 | 75 | 5 |
| 80+ | 881 | 8 | 1 | 911 | 4 | 0 | 935 | 9 | 0 |
| 20+ | 35440 | 269 | 36 | 29646 | 497 | 71 | 22658 | 714 | 107 |
| **Women** |  |  |  |  |  |  |  |  |  |
| 20-29 | 5767 | 34 | 8 | 4433 | 94 | 30 | 2414 | 84 | 25 |
| 30-39 | 7957 | 93 | 27 | 5881 | 137 | 40 | 3917 | 203 | 86 |
| 40-49 | 5947 | 123 | 37 | 6990 | 183 | 65 | 5425 | 230 | 83 |
| 50-59 | 5552 | 183 | 55 | 5709 | 248 | 68 | 5966 | 291 | 69 |
| 60-69 | 6069 | 306 | 72 | 4582 | 245 | 75 | 5085 | 303 | 91 |
| 70-79 | 4112 | 192 | 38 | 4074 | 235 | 53 | 3027 | 193 | 54 |
| 80+ | 1238 | 32 | 4 | 1327 | 45 | 9 | 1337 | 46 | 6 |
| 20+ | 36642 | 963 | 241 | 32996 | 1187 | 340 | 27171 | 1350 | 414 |

Figure S1: Prevalence of BMI defined (A) overweight (BMI 25-29.9 kg/m^2^) and (B) obesity (BMI ≥30 kg/m^2^) in men


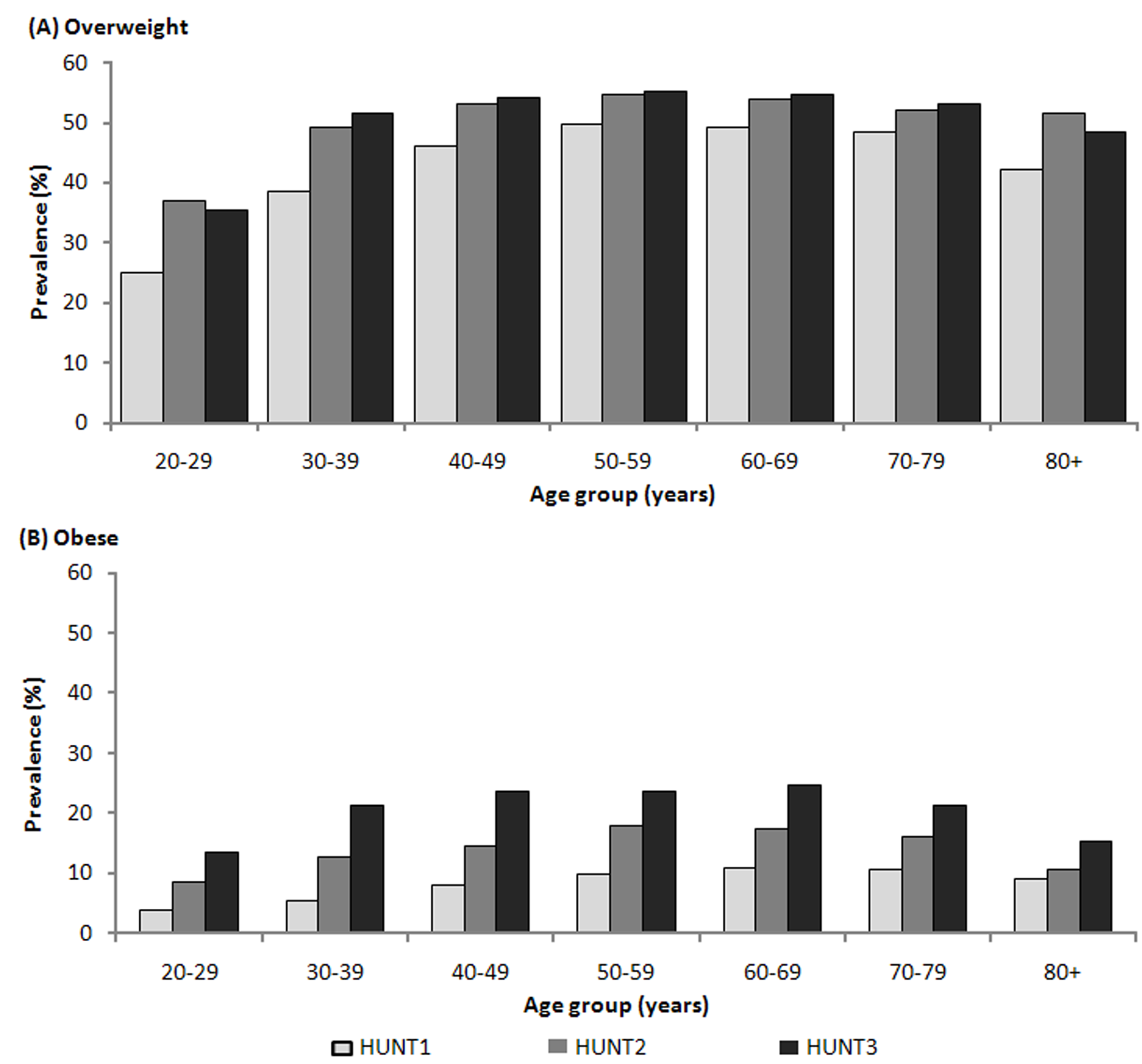


Figure S2: Prevalence of BMI defined (A) overweight (BMI 25-29.9 kg/m^2^) and (B) obesity (BMI ≥30 kg/m^2^) in women


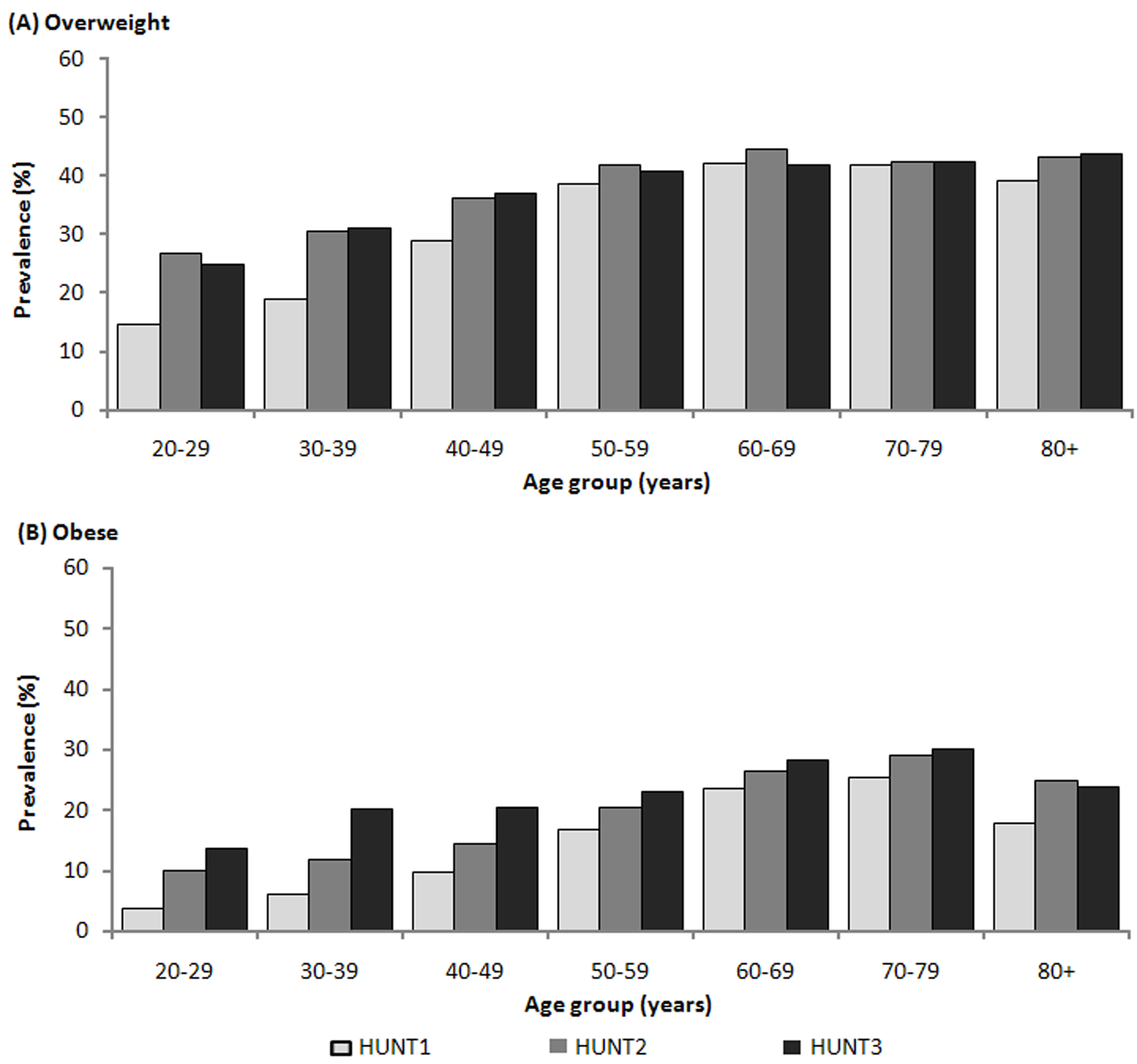


Figure S3: Prevalence of obesity (A) class I (BMI 30.0-34.9 kg/m^2^), (B) class II (BMI 35.0-39.9 kg/m^2^) and (C) class III (BMI ≥40.0 kg/m^2^) in men


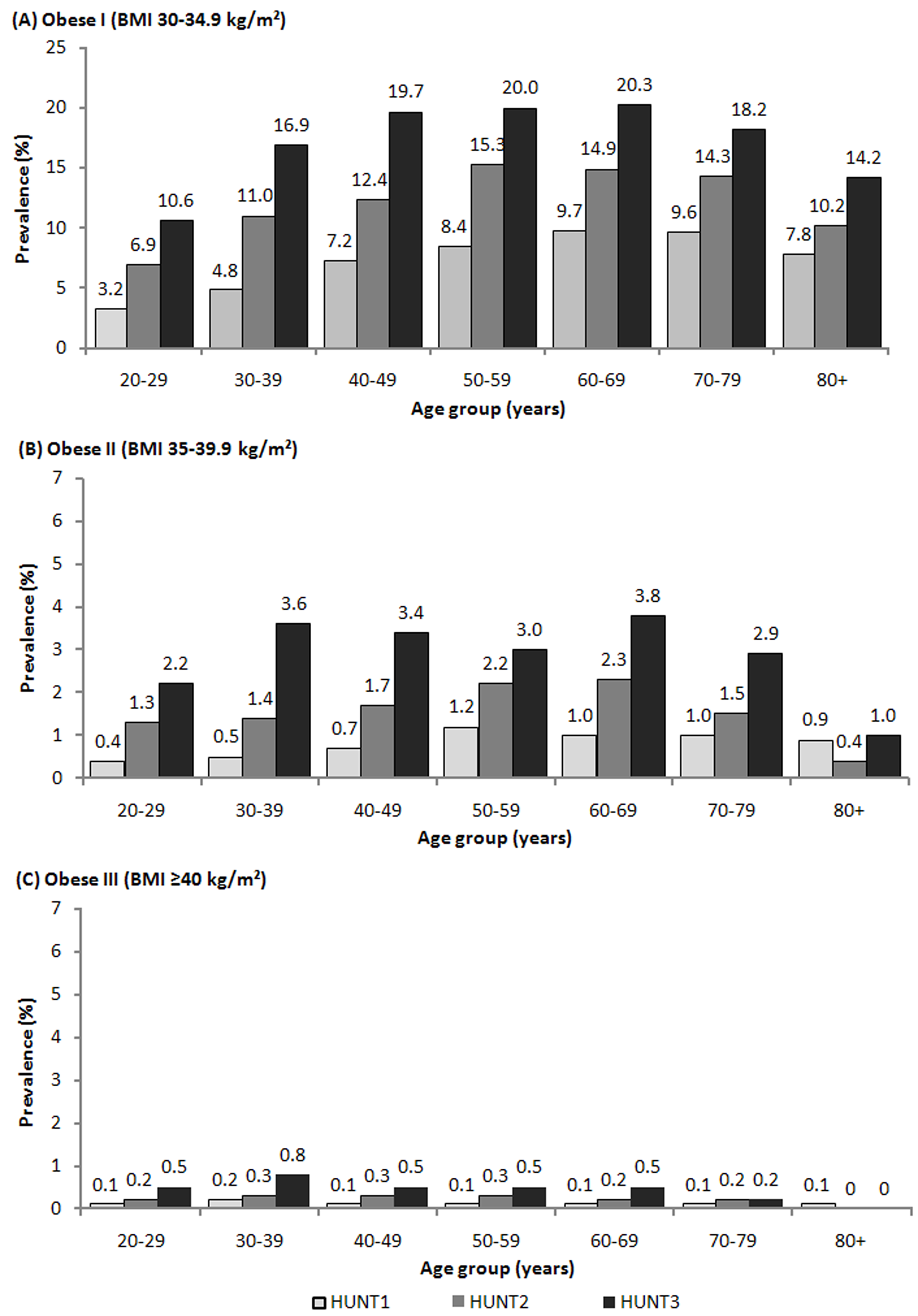


Figure S4: Prevalence of obesity (A) class I (BMI 30.0-34.9 kg/m^2^), (B) class II (BMI 35.0-39.9 kg/m2) and (C) class III (BMI ≥40.0 kg/m^2^) in women


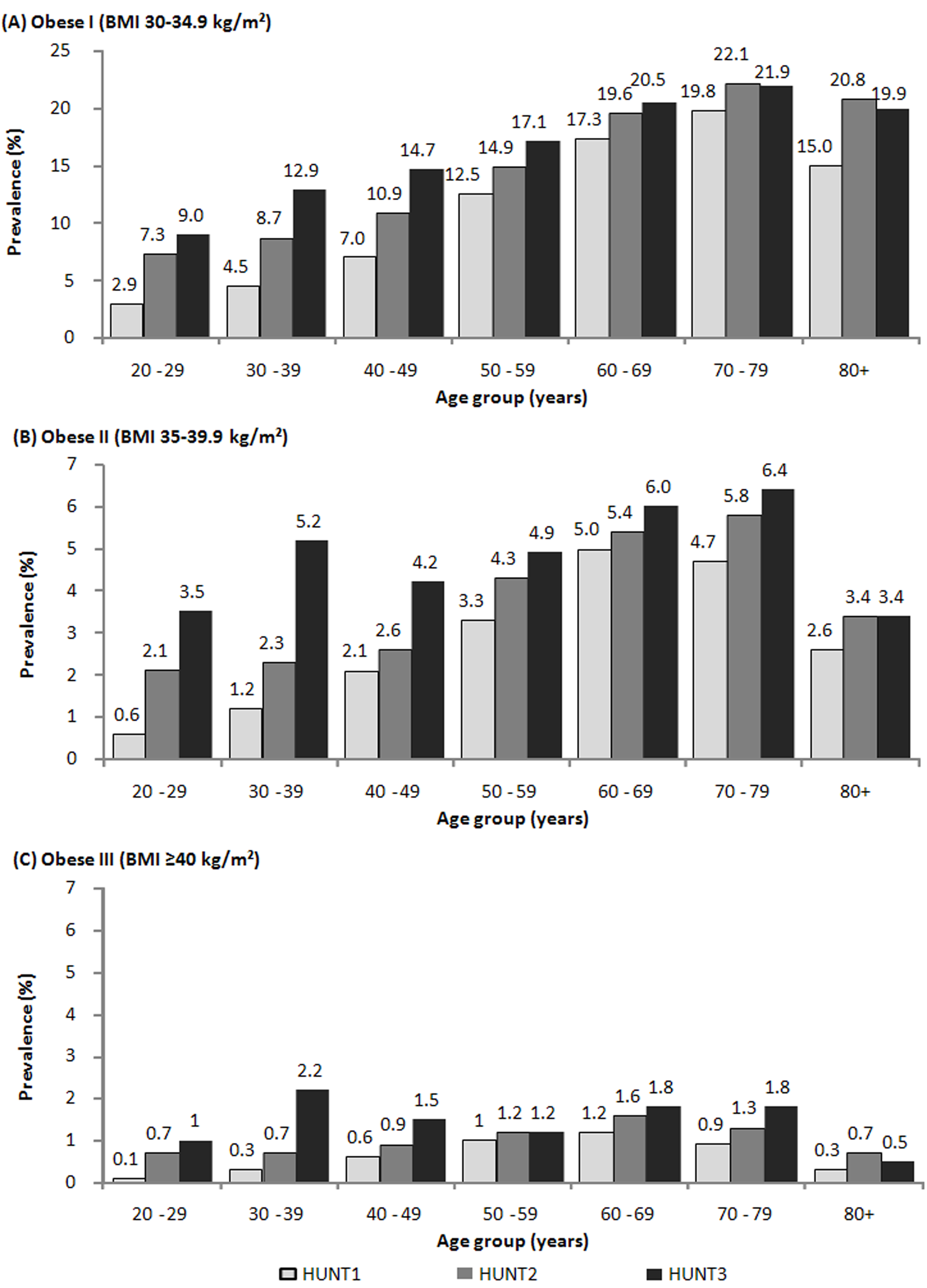


Figure S5: Prevalence of (A) abdominal overweight (WC 94.0-101.9 cm) and (B) abdominal obesity (WC >102.0 cm) in men


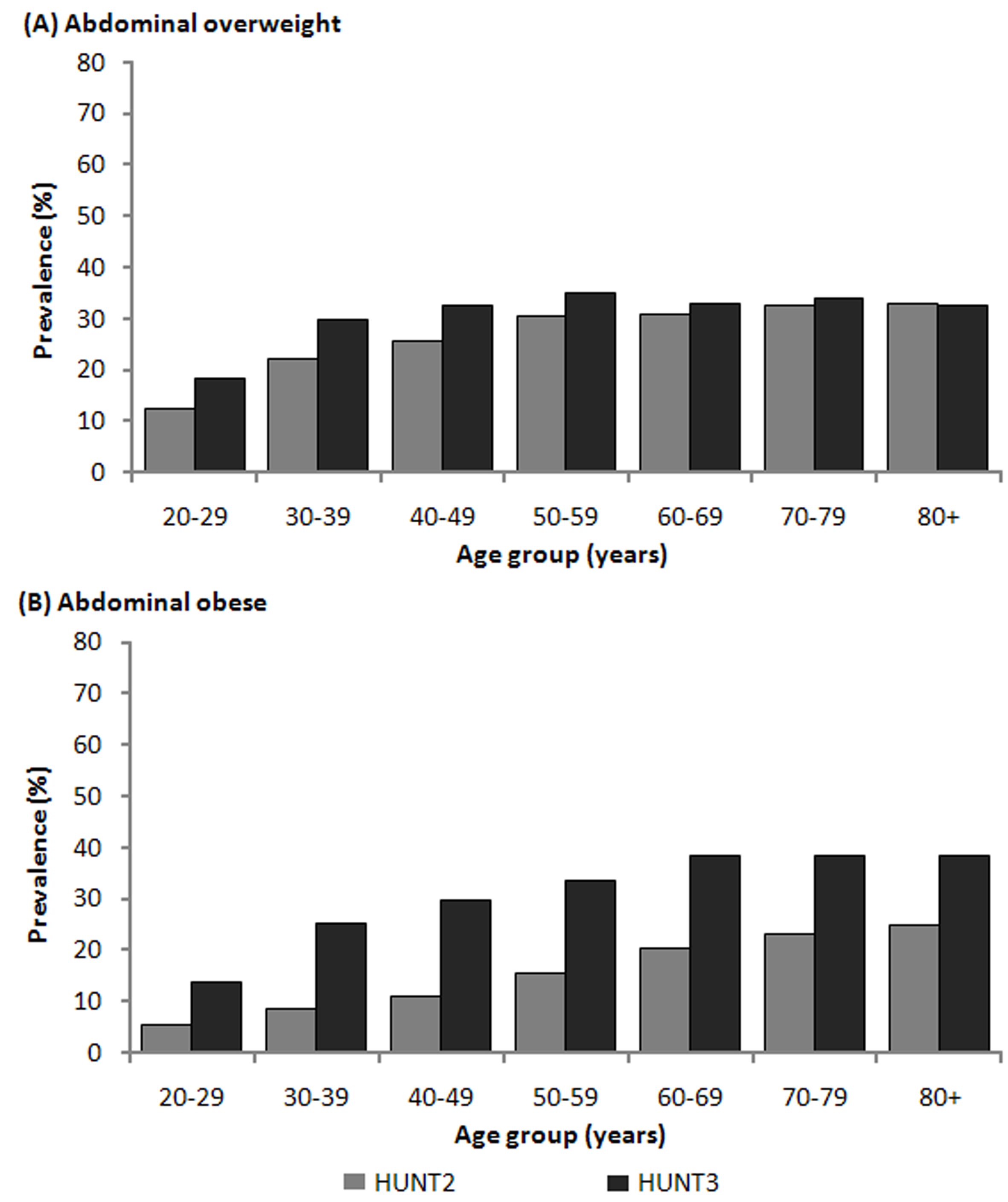


Figure S6: Prevalence of (A) abdominal overweight (WC 80.0-82.9 cm) and (B) abdominal obesity (WC> 88 cm) in women.


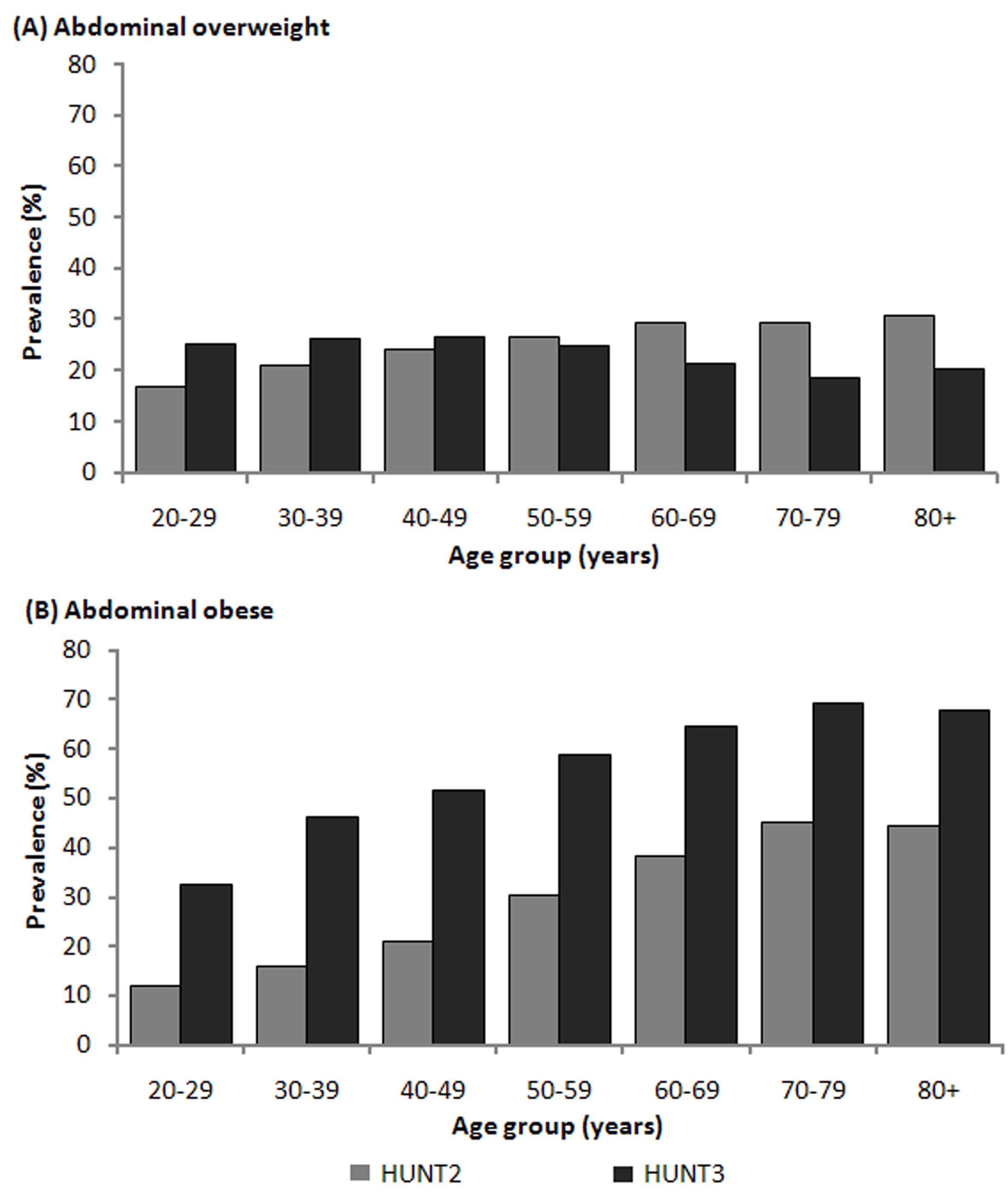

Supplement: Supplementary file 1 [file cob0003-0012-SD1.doc]
